# From digital museuming to on-site visiting: The mediation of cultural identity and perceived value

**DOI:** 10.3389/fpsyg.2023.1111917

**Published:** 2023-03-22

**Authors:** Yuanbing Deng, Xinhui Zhang, Bowen Zhang, Bei Zhang, Jing Qin

**Affiliations:** ^1^School of Journalism and Communication, New Media Research Institute, Zhengzhou University, Zhengzhou, Henan, China; ^2^Xinhua News Agency, Mu Qing Research Center of Zhengzhou University, Zhengzhou, Henan, China; ^3^College of Management, Shenzhen University, Shenzhen, China; ^4^Department of Mathematics, King's College London, London, England, United Kingdom

**Keywords:** online experience, perceived value, cultural identity, willingness to visit, consumer behavior, digital museum

## Abstract

**Introduction:**

Museums use digital resources to provide online services to the public, and a “digital museuming” boom has started. The mechanism of online museum visiting and its impact on willingness to visit on site has become an important issue of widespread concern. Therefore, based on the theory of presence and cognitive-emotional-behavioral theory, this paper introduces perceived value and cultural identity as mediating variables to explore the influence of the digital museuming experience on the willingness to visit on site from the audience’s perspective.

**Method:**

Questionnaires were distributed, using the snowball sampling method, and 429 valid questionnaires were returned.

**Results:**

The empirical test presents the following results: (1) virtual reality technology multi-dimensionally expands the digital museuming experience; (2) immersion, interaction and available experience promote willingness to visit on-site; (3) hedonic experience in the process of digital museuming cannot be ignored; and (4) perceived value and cultural identity play a mediating role.

**Discussion:**

User experience of visiting virtual museums, perceived value and cultural identity influence user willingness to visit museums in the field, but perceived value does not enhance the user’s cultural identity, maybe due to the inability of the online experience to increase the depth of the experience.

## 1. Introduction

Today, with advancements in digital technology, museums take on new forms of presentation. Virtual reality, artificial intelligence, the Internet, and smartphone-related services are becoming increasingly popular, and museums are becoming smarter as a result of this rapid wave of digital development (Proctor, 2011). Digital museums have increased their cultural heritage and content, fully utilizing their existing potential value (Choi and Kim, 2021).

As the United Nations Educational, Scientific, and Cultural Organization (UNESCO) (2020) reports, the outbreak of COVID-19 caused a large number of museums to temporarily close. The closure of museums due to COVID-19 and the massive decrease in visitors have had a huge impact on the museum industry (Choi and Kim, 2021), but the “stay-at-home economy” has become prevalent and pushed the development of digital business in many industries (Liao et al., 2023). The emergence of online services has met the demand for cultural consumption in such special times and, against this backdrop, digital museums have attracted public attention. The COVID-19 crisis has accelerated the digitization of museums themselves (Agostino et al., 2020), and more museums are adopting online exhibitions and services to provide the public with online experiences that transcend physical space and time constraints. China, for example, launched the “digital museuming at home” campaign, which received more than 26.76 million visitors in 2 days, including a peak of 174,000 visitors to the National Museum of China (Xinhua News Agency, 2020). Meanwhile, a beta version of an application, “digital museuming Dunhuang,” which was embedded in WeChat and QQ applications, was jointly launched by the Dunhuang Academy, People’s Daily New Media and Tencent. The number of visitors exceeded 5.7 million in 20 days (Art China, 2020). Similar services have been launched by world-renowned museums, such as the British Museum and the Louvre.

In the post-Covid-19 era, museums have not stopped digitizing, but instead sought to facilitate their offline experiences in an online format (Meng et al., 2022). Many users visit museums’ websites at home, or view exhibits online using virtual reality devices or live streaming. Not only can they visit museums anytime and anywhere, they can also enjoy the exhibits from multiple angles. Digital technology and virtual reality have huge potential for further development. This use of Internet platforms to visit exhibition halls and virtual museums is called digital museuming (Meng et al., 2022). This way of visiting museums breaks the space–time limits and brings unique experiences. For example, the real-time interaction of live streaming generates a sense of social presence and immersion (Chen and Liao, 2022). Hence, digital museuming generates psychological feelings and emotions, through the process of communication and interaction.

User experience impacts behavior intention. Zheng et al. (2022) note that destination live streaming impacts viewers’ travel intentions. In the post COVID-19 context, the integration of cultural tourism and digital tourism is particularly important, and we need to clarify how digital museums can effectively stimulate willingness to visit on site through digital means (Samaroudi et al., 2020). Given this background and the practical needs, this paper selects users who have participated in digital museuming on the Internet as research subjects to explore the impact of the digital museuming experience on museum visitors’ willingness to visit on site.

Drawing on presence theory and cognitive-emotional-behavioral theory, this study reveals two critical psychological mechanisms, perceived value and cultural identity, by which the digital museuming experience affects on-site visiting intention. The term “presence” is derived from the term “virtual presence” and refers to a feeling of immersion, embodied in a psychological state. It is generally accepted that presence is the psychological state in which participants feel they are in a virtual environment—a subjective feeling of being there rather than a real experience (Sanchez-Vives and Slater, 2005). Sense of presence is one of the most important advantages of digital tourism, as in destination live streaming and online commerce (Chen and Liao, 2022; Zheng et al., 2022). With the continuous development and practical application of virtual reality technology, the sense of presence felt by participants surrounded by and interacting with virtual environments has garnered attention. Presence theory emphasizes communication and interaction in online environments (Xu et al., 2021), and promotes an understanding of user experience. Meanwhile, cognitive-emotional-behavioral theory suggests that individual behavioral change has three stages. The theory notes that cognition affects user behavior. When people decide to visit a museum offline, their perceived online visiting experience impacts their intention and behavior. These two theories are useful and appropriate for explaining how digital museuming impacts on-site visiting.

This study is conducted to explore the impact of the digital museuming experience on users’ willingness to visit on site. Specifically, we attempt to answer the following research questions: (1) What are the factors that make up the digital museum experience? (2) Does the digital museuming experience affect willingness to visit museums on site? (3) What factors of the digital museuming experience affect willingness to visit museums on site? (4) Does the perceived value that audiences receive during digital museuming affect cultural identity? (5) Do audiences’ perceived values and cultural identities play a mediating role between the digital museuming experience and a visit? (6) Do audiences’ perceived values and cultural identities play a mediating role between the digital museuming experience and their willingness to visit?

## 2. Theoretical background and research hypothesis

### 2.1. Theoretical background

#### 2.1.1. Presence theory

Many scholars analyze the theory of presence and differences in individual experiences from a psychological perspective. Presence is a subjective feeling of “being there” in the digital context (Zahorik and Jenison, 1998; Schubert, 2009). Spatial presence as a cognitive feeling comes from the unconscious spatial cognition processes, which is the experience of being in the same space and time. When users are visiting online spaces, they not only perceive the “real” environment where they are physically present, but also the context defined by the multimedia environment. The perceived presence contributes to the user’s flow experience (Skadberg and Kimmel, 2004; Schubert, 2009). Zhang and Xu (2021) consider an individual’s presence to be a subjective experience. The degree of immersion is judged in two main ways: sensory immersion due to the characteristics of the virtual reality technology, degree of manipulation, immersion and interaction which directly affect the experience; and emotional arousal, with a higher level of emotional arousal making the immersive experience stronger (Güzel, 2014).

This paper uses “immersion” to refer to the immersive experience participants receive from the information in a virtual reality environment. When participants visit and explore a virtual destination, they can feel the presence of the destination, interact with local perceptions and communicate with real people in the virtual environment (Zahorik and Jenison, 1998). This serves as a bridge to help us understand the impact of online experiences on offline activities.

#### 2.1.2. Cognitive-emotional-behavioral theory

Cognitive-emotional-behavioral theory explains the occurrence of behavior, with the process of individual behavior change consisting of three stages, the cognitive, the emotional and the behavioral (Corstorphine, 2006; Zhou et al., 2022). Cognition is rational perception, including perceptions, beliefs, ideas and understanding, resulting from the individual’s exposure and experience. Emotions are mental states that develop on the basis of cognition and include judgments, emotions and feelings of attachment. Basic cognitive and emotional evaluation leads to behavioral intentions, tendencies, and preferences. Thus, individuals first form a cognition about something, and this cognition leads to an emotion about that thing, and eventually, guided by the cognition and emotion, they take actions (Corstorphine, 2006; Zhou et al., 2022). This theory is applicable to studies related to tourist behavior. Luo et al. (2021) construct a cognitive-emotional-behavioral structural equation model to explore the influence of poetry cognition and emotion evaluation on behavior intention from the perspective of tourists, based on cognitive-emotional-behavioral theory.

As a result of the COVID-19 outbreak, museums rely on advanced virtual reality technology to integrate existing cultural resources and conduct digital museuming activities for the general public in virtual space. After experiencing digital museuming, the public can measure the time and effort spent on digital museuming to generate perceived value, which is the basis of behavioral intention. Cultural identity is generated and an emotional evaluation of the museum as a whole is made, and this emotional empathy determines their willingness to visit the site.

### 2.2. Research hypotheses

#### 2.2.1. Digital museuming experience, perceived value and cultural identity

The audience takes in the virtual sense of the digital museuming experience through the perceptions of their five senses and their own experiential process. As Alba and Hutchinson (1987) argue, the experience not only familiarizes consumers with the product and deepens their unique impressions but also creates brand associations, thus creating a strong personal connection with the brand. Unlike offline experiences, the sensory stimulation of the virtual environment makes it easier for consumers to understand the product, and research indicates that consumers who have an online experience are more likely to have a strong perceived value than those who only have an offline experience.

Many studies have been conducted into the dimensions of the tourism experience. Otto and Ritchie (1995) summarize six sub-dimensions, novelty, hedonics, interaction, stimulation, security and comfort, which constitute an overall service experience. Otto and Ritchie (1996) view tourism as a service industry, and emphasize the subjective feelings experienced by customers when they consume and evaluate services. The dimensionality of the service experience in tourism is composed of hedonics, peace of mind, involvement and recognition. Kim et al. (2012) develop a scale to measure tourism experiences, including hedonism, refreshment, local culture, meaningfulness, knowledge, involvement and novelty. In the digital era, the culture and tourism industry constantly tries to develop new tourism experiences with virtual reality and other digital technologies. He et al. (2018) argue that virtual reality experiences, in the context of digital museums, include visual appeal, entertainment, enjoyment, and escapism. Technology is one of the key factors that determines the virtual tourism experience. Perez-Marcos (2018) explores the main components of virtual reality experience, immersion, interaction, sensorimotor contingencies and illusions. A virtual reality-based model of experience in museums is constructed by Trunfio and Campana (2020), dividing museum visitor experience into seven dimensions, information, customization, format, usability, information saving, interaction and sense of experience. Wu et al. (2020) introduce the virtual reality experience into the field of tourism and propose that virtual tours include immersion, interaction, usability and illusion. When users access a digital museum, they are immersed in the environment and see the exhibits through technology facilities. At the same time, users can operate digital devices for interaction and interact with each other, resulting in a rich visiting experience. Based on the literature, combined with the characteristics of visiting digital museums, the digital museuming experience is divided into four dimensions: cognitive immersion experience, interactive experience, available experience and hedonic experience. Cognitive immersion is a mental state in which people who are interested in something become completely immersed. Interactive experience is an important part of obtaining a high degree of immersion in virtual museums, which can be divided into several types, personal roaming, human-computer interaction and interaction among visitors. Available experience refers to the effectiveness, efficiency and satisfaction of people in terms of achieving their goals. It refers to how users feel and whether, and to what extent, their visiting needs are met when they visit a digital museum. Hedonic experience refers to the emotions of relaxation and enjoyment evoked by roaming in digital museums, based on certain sensory experiences and emotional satisfaction.

Because digital museuming activities are inherently immersive (Thomas and Carey, 2005; Jin et al., 2015; Errichiello et al., 2019), interactive (Shipps and Phillips, 2013), available (Flavián et al., 2006), and hedonic (He et al., 2018), this study classifies digital museuming experience into cognitive immersion experience, interactive experience, available experience and hedonic experience. It is expected for the audience to produce corresponding subjective feelings and form their own unique perceived values when participating in such a unique environment as digital museuming. Based on the above, the following hypotheses are proposed:

*H1*: The experience of digital museuming positively affects the perceived value.

*H1a*: The cognitive immersion experience in digital museuming positively influences the perceived value.

*H1b*: The interactive experience in digital museuming positively influences the perceived value.

*H1c*: The available experience in digital museuming positively influences the perceived value.

*H1d*: The hedonic experience in digital museuming positively influences the perceived value.

Individual subjectivity in tourism has received much attention. There is a close relationship between identity, as an influencing factor of the tourism experience, and individual subjectivity. In tourism activities, a positive tourism experience can enhance the perceived value of the tourist, which in turn reinforces cultural identity towards the destination (Agarwal and Karahanna, 2000). Using both quantitative and qualitative research methods to examine the experiences of tourists at sites associated with natural or man-made disasters or atrocities, Kang et al. (2012) show a significant positive correlation between tourism experience and personal gain.

Culture is the essence of a place and is symbolic of the spiritual dimension. Cultural identity reflects feelings towards a certain culture and the sense of belonging to a culture (Pan et al., 2019; Gao et al., 2020; He and Ai, 2021). It is the process of integrating culture into an individual’s self-concept, which reflects not only their identification with the culture but also their perceptions of a set of elements associated with the culture. The formation of cultural identity is a long and complicated process and develops gradually in social surroundings, encompassing the recognition of religion, history, customs and society (Keillor et al., 1996; Altugan, 2015). From the perspective of cultural attitudes, cultural identity is a key attribute that influences the psychology and behavior of tourists. Thus, cultural identity reflects the emotional side of the tourist, emphasizing their cultural emotions regarding the destination, which is an important factor influencing tourism behavior (Tian et al., 2020). Specifically, cultural identity can enhance the initiative of the tourist and is a potential factor guiding actual behavior. The value of a destination is more easily accepted and appreciated by tourists if their cultural identity is evident, and is a priority factor for a positive experience. Museums have a certain cultural carrying capacity, and tourists tend to develop a sense of belonging when visiting and touring museums, which in turn influences their cultural identity.

Accordingly, this study argues that cultural identity is a positive emotion, directed towards culture, that audiences generate during their digital museuming. With the development of the information society, museum tourism gradually integrates with digitalization, while creating its own characteristics, and continues to show humanistic meaning, enhancing audiences’ cultural identity with the museum. Cultural identity, to a certain extent, relates to what individuals gain at the end of the tour, and this also applies to the relationship between the digital museuming experience and cultural identity. Digital museuming provides a new way for audiences to experience history and culture through immersive and interactive virtual cultural spaces that communicate the cultural connotations of museums, enhancing people’s sense of cultural identity. Based on the above, the following hypotheses are proposed:

*H2*: The experience of digital museuming positively influences cultural identity.

*H2a*: The cognitive immersion experience in digital museuming positively influences cultural identity.

*H2b*: The interactive experience in digital museuming positively influences cultural identity.

*H2c*: The available experience in digital museuming positively influences cultural identity.

*H2d*: The hedonic experience in digital museuming positively influences cultural identity.

#### 2.2.2. Digital museuming experience and willingness to visit on site

The virtual reality environment created through digital museuming activities can provide audiences with first-hand experience similar to on-site tourism, prompting potential audiences to learn real-world information about the destination. Li et al. (2021) select groups of people with virtual tourism experience as subjects and explore the transformation mechanisms between virtual tourism experiences and on-site tourism behavior. The importance of the virtual tourism experience is increasing, which not only provides potential tourists with a digital sensory experience but also enables tourists to feel the destination in advance, which helps them make decisions and affects their willingness to visit on site (Cheng and Huang, 2022). As an important technological tool, digital museuming can be seen as a new counterpart to the willingness to visit museums. Visitors who have had a digital museuming experience focus more on the content of the experience itself, which, to some extent, provides them with the possibility to visit museums in the field. Based on the above, this study proposes the following hypotheses:

*H3*: The digital museuming experience positively influences the willingness of the audience to visit on site.

*H3a*: The cognitive immersion experience in digital museuming positively influences audience willingness to visit on site.

*H3b*: The interactive experience in digital museuming positively influences audience willingness to visit on site.

*H3c*: The available experience in digital museuming positively influences audience willingness to visit on site.

*H3d*: The hedonic experience in digital museuming positively influences audience willingness to visit on site.

#### 2.2.3. Perceived value, cultural identity and willingness to visit on site

The study of perceived value began in the 1980s, along with the advent of the experience economy, when companies began to pay attention to customers’ consumption experience. Zeithaml (1988) defines perceived value as the consumer’s overall evaluation of the utility of a product or service based on the perceptions of what was gained and given; a trade-off between perceived benefits and perceived costs. In the field of management research, perceived value is often seen as a subjective construct that changes with time and consumer culture. Babin et al. (1994) state that the general definition of value is the consumer’s perception of the subjective value of some activity or object, taking into account all the net benefits and consumption costs. Nowadays, scholars explore the connotations of perceived value, arguing that tourism destinations demonstrate their value and unique service (Gallarza and Saura, 2006). Although perceived value is abstract, it can still be perceived. Therefore, this study defines perceived value as a subjective feeling that audiences have after visiting a digital museum in person, based on the time and effort they spend.

From past literature, it is known that there is a significant relationship between perceived value and consumers’ behavioral intentions. Meng (2018) studies the relationship between perceived value and consumer behavior in virtual brand communities, and classifies perceived value into four dimensions, psychological value, functional value, entertainment value and social value, concluding that all four dimensions have a positive correlation with consumers’ behavioral intentions. Extended to the field of tourism, the perceived value of virtual tourism has a significant impact on actual behavioral intentions (Farivar and Wang, 2022). In the process of digital museuming, audiences derive perceived value, which can influence their willingness to visit on site. Accordingly, this study formulates the following hypothesis:

*H4*: The perceived value following digital museuming positively influences willingness to visit on site.

Based on the conceptual definition of cultural identity, this study argues that it reflects positive emotions toward a culture. Many scholars focus on tourists’ emotions and behavioral intentions and analyze the relationship between the two empirically, concluding that tourist emotion positively influences behavioral intention (Chen et al., 2022). Thus, if visitors develop a strong sense of belonging in the process of digital museuming, they will want to visit their favorite museums in person to experience the real surroundings, facilities and cultural atmosphere. At the same time, they will be willing to recommend the museum to their friends and family. Based on the above, the study proposes the following hypothesis:

*H5*: Following digital museuming, cultural identity positively influences willingness to visit the site in person.

Many scholars study the relationship between perceived value and brand identity, local identity, community identity, cultural identity, etc. For example, Han (2020) uses an empirical research method to specifically analyze the perceived value of virtual community members and brand identity based on the stimulus-organism-response model, and the results show that the perceived value of virtual community members has a significant positive impact on brand identity. Kaur et al. (2020) make a similar argument. In the process of digital museuming, audiences can experience public services and the uniqueness of museum culture, and gain pleasure and emotional satisfaction, deepening their personal sense of identity and belonging to museum culture. Based on the above, the study proposes the following hypothesis:

*H6*: Perceived value positively influences cultural identity following digital museuming.

#### 2.2.4. The mediating role of perceived value and cultural identity

In the field of cultural heritage, studies use perceived value as a mediating variable in the tourism experience model, confirming its mediating role and suggesting that positive evaluation can promote positive perceptions of cultural heritage and enhance the experience of Nature resource management culture. Gao et al. (2020), based on theory related to ethnic nature, take cultural identity as a mediating variable to explore its role in ethnic contact, using a questionnaire method. The results show that cultural identity about own ethnicity plays a mediating role between ethnic contact and ethnic essentialism (Zhang et al., 2021). Perceived value and cultural identity play mediating roles between audience experiences and behavior. Satisfied visitors revisit and recommend destinations to others (Prayag and Ryan, 2012). Hence, after experiencing digital museuming, audiences develop their perceptions of value and cultural identity toward the museum, which in turn influences their intention to visit. Based on the above, this study proposes the following hypotheses:

*H7*: Perceived value mediates the relationship between digital museuming experience and willingness to return to the site following digital museuming.

*H7a*: Following digital museuming, perceived value mediates cognitive immersion experience and willingness to visit on site.

*H7b*: Following digital museuming, perceived value mediates interactive experience and willingness to visit on site.

*H7c*: Following digital museuming, perceived value mediates available experience and willingness to visit on site.

*H7d*: Following digital museuming, perceived value mediates hedonic experience and willingness to visit on site.

*H8*: Following digital museuming, cultural identity mediates digital museuming experience and willingness to visit on site.

*H8a*: Following digital museuming, cultural identity mediates cognitive immersion experience and willingness to visit on site.

*H8b*: Following digital museuming, cultural identity mediates interactive experience and willingness to visit on site.

*H8c*: Following digital museuming, cultural identity mediates available experience and willingness to visit on site.

*H8d*: Following digital museuming, cultural identity mediates hedonic experience and willingness to visit on site.

**Figure 1:**
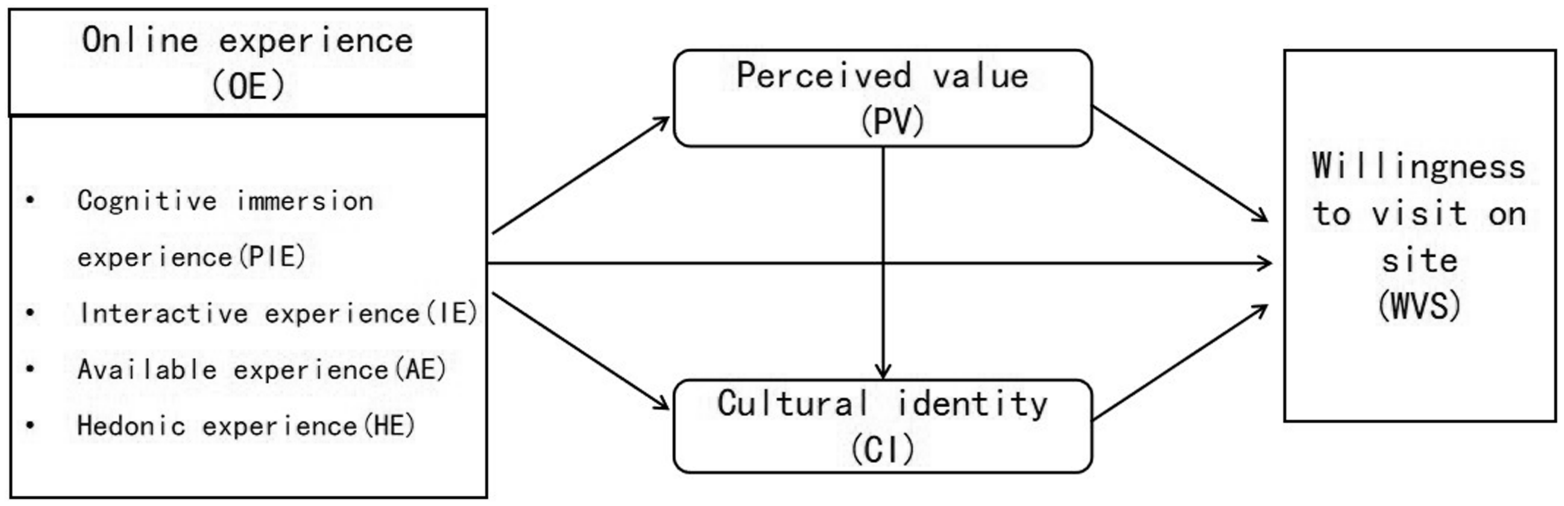
Theoretical model.

By exploring the relevant theoretical foundations and reviewing the existing literature, the theoretical research model is finally constituted based on the logical relationship and hypothesis analysis of four elements: the digital museuming experience, perceived value, cultural identity and willingness to visit on site. This paper explores the influence of digital museuming experience on willingness to visit on site, taking digital museuming experience as the antecedent variable from the theory of presence. Meanwhile, the research model is constructed based on cognitive-emotional-behavioral theory, with perceived value following a digital museuming experience as the cognitive element, cultural identity as the emotional element, and willingness to visit on site as the behavioral element. The theoretical model of the study is shown in Figure 1.

## 3. Research design

### 3.1. Data collection and sample characteristics

This study selects users who have participated in digital museuming activities and therefore have digital museuming experience as the research subjects. Questionnaires were distributed through one-to-one electronic contacts, WeChat groups, WeChat Moments circles, Weibo-related topics, the Super Talk community, the Douban museum group, etc (Zeng and Zhu, 2021). The period of study is from September 15 to November 30, 2021. The sampling techniques are the non-probability judgmental sampling method and snowball method. A total of 536 questionnaires were distributed, and 475 were returned. In order to improve the quality of the questionnaires, the researcher screened them in terms of response level and response length, and found that the overall completion level was good. After analyzing the initial data, 46 invalid questionnaires with response times of less than 90 s were excluded, leaving 429 valid questionnaires; an effective rate of 90.32%.

Among the survey respondents, women account for 55%, and they are mainly young or middle-aged (18–35 years old accounting for 76.7%), possess a higher education level (a bachelor’s degree or above accounting for 67.6%). More than two-thirds of respondents have rich digital museuming experience, and nearly half the respondents have been digital museuming for a long time, which reflects the attractiveness of digital museuming (see Table 1).

**Table 1:** Demographic characteristics of the respondents.

Variable	Item	Frequency	Percentage (%)
Gender	Male	193	45
	Female	236	55
Age	≤18	22	5.1
	18–25	185	43.1
	26–35	144	33.6
	36–45	54	12.6
	≥45	24	5.6
Education	Junior high school or below	8	1.9
	High school	47	11
	Junior college	84	19.6
	Undergraduate	213	49.7
	Postgraduate or above	77	17.9
Frequency of digital museuming	Once	156	36.4
	2–3 times	187	43.6
	4–5 times	60	14
	6 times or above	26	6.1
Duration of digital museuming	<10 min	128	29.8
	10–30 min	172	40.1
	30 min to 1 h	67	15.6
	1–2 h	47	11
	>2 h	15	3.5
	Total	429	100

### 3.2. Measurements

In this empirical study of digital museuming, the experience scale is based on Shipps and Phillips (2013) and Jin et al. (2015), modified to incorporate the characteristics of digital museuming, with 16 items. The perceived value scale is based on Gallarza and Saura (2006) and Meng (2018), and consists of 4 items. The cultural identity scale is based on Pan et al. (2019) and Tian et al. (2020), and uses 5 items. The willingness to visit scale is based on Prayag and Ryan (2012), and consists of 3 items. All items are measured on a 5-point Likert scale, where 1 indicates “strongly disagree” and 5 indicates “strongly agree.”

## 4. Data analysis and results

### 4.1. Reliability and validity analysis

Cronbach’s alpha coefficient is a common measure of reliability, the value of which is proportional to consistency and reliability. A reasonable alpha coefficient of 0.7 is required for scale reliability, and the closer the coefficient is to 1, the higher the reliability. Meanwhile, convergent validity and discriminant validity together constitute structural validity. A validation factor analysis is conducted using AMOS 24.0 software to test the structural validity of the scale. A standardized factor loading greater than 0.6, a composite reliability (CR) value greater than 0.7, and an average variance extracted (AVE) value greater than 0.5 indicate good convergent validity of the scale. The results of the reliability and validity analysis of each study variable are shown in Table 2. When the correlation coefficient of each variable with the other variables is no greater than the square root of the AVE of that variable, it indicates that the scale has good convergent validity, as shown in Table 3.

**Table 2:** Reliability and validity analysis.

Variable	Item	Standardized factor loading	AVE	CR	Cronbach’s α
Cognitive immersion experience	Digital museuming has sparked my curiosity	0.884	0.786	0.936	0.936
	Digital museuming makes me feel like I’m there	0.881			
	When I am in digital museuming, I become so involved that I forget everything around me	0.879			
	Digital museuming frees me from reality, from my daily life	0.901			
Interactive experience	When I am in digital museuming, I am free to choose what I want to see	0.823	0.715	0.909	0.909
	When I am in digital museuming, I am able to get a positive response to every step of my operation	0.847			
	When I am in digital museuming, I can interact and give feedback in real time	0.868			
	When I am in digital museuming, I can feel the connection between people and do not feel alone	0.845			
Available Experience	The interface of digital museuming is clearly guided and everything is easy to understand	0.883	0.773	0.932	0.932
	The operation of digital museuming is very simple, even for the first time, I was able to operate it skilfully	0.885			
	I can easily find what I need in the process of digital museuming	0.870			
	When I am in digital museuming, I think the content is rich and the structure is well set	0.879			
Hedonic experience	I am very happy to have the experience of digital museuming	0.873	0.759	0.927	0.926
	The process of digital museuming makes me feel at ease.	0.878			
	In my opinion, I really like digital museuming	0.867			
	I enjoy the digital museuming process	0.867			
Perceived value	The digital museuming experience has taught me a lot and has met my expectations	0.820	0.676	0.893	0.923
	I find the digital museuming experience very interesting and enjoyable	0.817			
	I am very satisfied with the time and effort I put into digital museuming	0.819			
	I think the digital museuming experience is well worth it	0.833			
Cultural identity	After digital museuming, I have a better understanding of museum culture than before	0.852	0.701	0.921	0.947
	During the process of digital museuming, I develop a strong sense of national pride and cultural self-confidence	0.823			
	I would like to spend more time in digital museuming if I can	0.836			
	After digital museuming, I would like to be involved in activities related to museum culture	0.826			
	After digital museuming, I will discuss museums with people who have similar cultural backgrounds as me	0.848			
Willingness to visit on site	After digital museuming, I have the willingness to visit this museum on site	0.875	0.743	0.896	0.924
	After digital museuming, I will recommend my friends and relatives to visit this museum on site	0.834			
	Even after visiting the digital museuming, I have the desire to visit again	0.875			

**Table 3:** Distinct validity.

	AVE	Cognitive immersion experience	Interactive experience	Available experience	Hedonic experience	Perceived value	Cultural identity	Willingness to visit on site
Cognitive immersion experience	0.786	0.887						
interactive experience	0.715	0.517	0.846					
Available Experience	0.773	0.625	0.502	0.879				
Hedonic experience	0.759	0.524	0.532	0.453	0.871			
Perceived value	0.676	0.547	0.622	0.549	0.515	0.822		
Cultural identity	0.701	0.646	0.577	0.576	0.575	0.572	0.837	
Willingness to visit on site	0.743	0.599	0.582	0.550	0.485	0.599	0.672	0.862

### 4.2. Model and direct path test

#### 4.2.1. Model fit analysis

The overall goodness-of-fit of the model is tested using AMOS 24.0, before the hypothesis testing of the model is performed. The fit of the model is analyzed by maximum likelihood estimation (MIE), and the fit indicators are: χ^2^/df value, root mean square error of approximation (RMSEA), goodness of fit (GFI), normed fit index (NFI), incremental fit index (IFI), Tucker Lewis index (TLI), and other indicator values. When the sample is large, the χ^2^/df criterion can be relaxed from 1–3 to 1–5; RMSEA below 0.05 indicates an excellent fit, and between 0.05 and 0.08 indicates an acceptable fit; the values of CFI, NFI, IFI, and TLI are between 0 and 1, and values above 0.9 represent an excellent fit; the parsimony normed fit index (PNFI) and parsimony comparative fit index (PCFI) values are above 0.6, representing an acceptable fit. The results of the theoretical model fit indexes are shown in Table 4.

**Table 4:** Model fitness index values.

Index	Evaluation criteria	Research model
χ^2^	The smaller the better, *p* < 0.05	1067.297 (*p* = 0.000)
χ^2^/df	1–5	3.144
RMSEA	<0.08	0.071
NFI	>0.9	0.909
IFI	>0.9	0.936
TLI	>0.9	0.928
CFI	>0.9	0.936
PNFI	>0.6	0.806
PCFI	>0.6	0.830

#### 4.2.2. Direct path test

The structural equation model is constructed using AMOS 24.0 and tested to obtain the standardized path coefficient (estimate), standard error of the estimate (SE), critical ratio (CR), and *p*-value. The standardized path coefficient indicates the amount of change in Y when X changes by one standard deviation, and the correlation between the variables is determined by the absolute value of the path coefficient, which is proportional. The t-value in the t-test is the critical ratio, and the p-value is the degree of significance of the path. When the *p* value is less than 0.05, the hypothesis is generally considered valid. From the results of the path test, it can be seen that hypotheses H3d and H6 are not valid. Hypotheses H1a, H1b, H1c, H1d, H2a, H2b, H2c, H2d, H3a, H3b, H3c, H4, and H5 are valid. The specific values are shown in Table 5.

**Table 5:** Path test results.

NO.	Hypothesized paths	Estimate	SE	CR	*P*
H1	PV <− PIE	0.150	0.033	4.502	***
	PV <− IE	0.384	0.041	9.458	***
	PV <− AE	0.203	0.035	5.749	***
	PV <− HE	0.154	0.036	4.300	***
H2	CI <− PIE	0.281	0.034	8.323	***
	CI <− IE	0.190	0.042	4.475	***
	CI <− AE	0.155	0.035	4.431	***
	CI <− HE	0.208	0.035	5.885	***
H3	WVS <− PIE	0.153	0.040	3.812	***
	WVS <− IE	0.184	0.048	3.821	***
	WVS <− AE	0.086	0.039	2.185	*
	WVS <− HE	−0.015	0.040	−0.366	0.714
H4	WVS <− PV	0.219	0.063	3.488	***
H5	WVS <− CI	0.380	0.064	5.921	***
H6	CI <− PV	0.111	0.057	1.951	0.051

*P < 0.05, ***P < 0.001.

#### 4.2.3. Intermediary effect test

The demographic variables are used as control variables, and a bootstrap mediation test is applied using PROCESS software. The results are shown in Supplementary Appendix Table S1 in the appendix. When the model takes cognitive immersion experience, interactive experience and available experience as independent variables, the coefficients of each path are positive and significant. When the model takes hedonic experience as the independent variable and willingness to visit as the dependent variable, the effect of hedonic experience on willingness to visit a site is not significant, while the coefficients of other paths are positive and significant. The total effect, direct effect and indirect effect are tested.

From Supplementary Appendix Table S2, it can be seen that the total effect exists in paths 1, 2, 4, 5, 7, 8, 10, and 11, as well as an indirect effect, which indicates the mediating effect of the paths. The upper and lower confidence interval (CI) limits for the direct effects of paths 10 and 11 both contain 0, indicating full mediation. In the other paths, the upper and lower CI limits of the direct effect do not contain 0, indicating that the direct effect exists and is partially mediated. Based on the relative effect values, it can be concluded that the indirect effect plays a larger role than the direct effect, so hypotheses H7 and H8 are valid.

## 5. Conclusion and discussion

The primary goal of this paper is to investigate how the user experience of online access to virtual museums influences offline willingness to visit. The empirical results show that perceived value and cultural identity act as mediating variables connecting the user experience of online access to virtual museums with offline willingness to visit. In addition, the perceived value generated from the online experience fails to promote cultural identity with the museum. This study contributes to theoretical research in the field of user museum access by establishing a link between virtual access and willingness to visit on site.

### 5.1. Cloud touring museums: Which online experience better promotes consumer willingness to visit on site?

Firstly, based on cognitive-emotional-behavioral theory, this paper constructs a theoretical framework of how online experience affects offline willingness to visit museums, expanding the scope of application of the psychological cognitive-emotional-behavioral theory. The findings show that the more positive the cognitive experience of visiting a virtual museum, the more positive affective evaluations are generated, which in turn promote willingness to visit museums on site. This supports the dynamic cognitive-emotional-behavioral process in museum-visiting behavior. The findings confirm those of Marty (2007) and He et al. (2018). Studying the user experience of visiting museum websites, Marty (2007) notes that users often access information online, that helps them visit physical museums. He et al. (2018) explore the role of virtual reality technology in enhancing the museum experience and increasing the willingness to visit, taking an experimental approach. The findings show the relationship that exists between in-person and virtual museums, and provide strategies to support the changing information needs and experiences of museum visitors before and after visits. Virtual museums use their collections and cultural resources creatively to inspire people to experience, explore and develop ideas about tourism (Aurindo and Machado, 2016).

Secondly, users’ experiences of visiting virtual museums, perceived value and cultural identity influence their willingness to visit museums in the field, and perceived value and cultural identity play a mediating role in the willingness to visit museums in the field. The results show that cognitive immersion experience, interactive experience, available experience and hedonic experience all have a significant positive effect on museum travelers’ perceived value and cultural identity. Additionally, cognitive immersion, interactive experience and available experience have a significant positive influence on visit intention. Virtual museums use advanced virtual reality technology to creatively recreate museum scenes and give users a near-real experience. Users can interact with each other in real time during this process, which increases their willingness to visit on site. However, the empirical results show that the promotion of willingness to visit a site by hedonic experience is not fully supported, and this result is not consistent with the theory. To explore the reasons behind this, some of the subjects who participated in the questionnaire were invited to conduct additional interviews. The interviews revealed that, because most current virtual museums have more conventional visiting scenarios, the entertainment features of the museums are less appealing, and the hedonic experience of the users is not fully satisfied. Therefore, it is not possible to promote a positive willingness to visit on site among these users. In the interviews, the users were asked whether they had any regrets about digital museuming, one said:

*“There is a lack of entertainment activities, and the mood of entertainment is not satisfied, so there is not much joy in the process of digital museuming. Digital museuming is new to me, but it’s a bit boring to just visit and browse. But if digital museuming could be developed with more features and interesting activities, I believe the time spent by everyone in the digital museuming process would become a little longer.”*

The findings suggest that, with the continuous development of Internet and virtual reality technology, digital museuming could attract audience attention through immersive experiences (naked eye 3D, etc), optimizing the user’s digital museuming experience, and promoting willingness to visit on site.

Thirdly, the perceived value generated by visits to virtual museums does not enhance cultural identity, which is inconsistent with the theoretical hypothesis. As a spatial carrier of historical civilization, museums contain rich cultural resources, and virtual museums use the Internet and virtual reality technology to reproduce traditional culture (Xiao and Deling, 2016). However, as virtual museum development in China is in its infancy, the lack of perfect technology leads to a lack of depth in the virtual museums’ cultural resources and other problems not yet deeply explored in the cultural values hidden in the museum. In the future, virtual museums should give full play to the advantages of virtual reality, not only in terms of technological and morphological innovation but also in terms of cultural communication (Russo et al., 2006).

### 5.2. Enhancing the online experience and deepening cultural integration: Implications for museums

The digital preservation and dissemination of classical culture is an important trend in cultural transmission, and the emergence of virtual museums indicates the impact of technology on human behavior and the cultural tourism industry (Rafferty and Pad, 2017). This study reveals how users’ online digital experiences affect their offline willingness to visit museums, providing a deeper understanding of the mechanisms underlying user behavior at the theoretical level, on the one hand, and bringing insight to the practices of the cultural tourism industry on the other.

Firstly, the cultural tourism industry should accelerate digital construction in order to create a virtual cultural communication space which would attract participants. Compared to traditional museum tourism, virtual museums break barriers of time and space to present in a different way. With artificial intelligence, holograms and other technologies a virtual reality environment can be created, and this has become a development trend for museums. The Palace Museum has built the virtual Forbidden City, recreating royal life scenes and tourist routes, and simulating interaction between ancient and contemporary visitors. Users can easily enjoy the palace architecture and interact with other visitors. However, the current virtual museum construction is insufficient and does not reach spiritual or cultural levels. In the post-epidemic era, the social significance of the Internet is becoming more prominent, and virtual cultural spaces are gradually becoming important for cultural activities.

Secondly, the cultural tourism industry should pay attention to users’ online access experiences and carry out special activities to enhance the pleasure of the digital museuming experience. In the future, virtual museums should combine cultural communication with the trends of the times to continuously attract attention and interest. For example, the Suzhou Museum and Alipay have launched an augmented reality treasure hunt, which allows visitors to experience the traditional culture of Suzhou through modern technology, and a new type of museum visit. Users can learn about the cultural relics by simply taking out Alipay and sweeping it. These series of activities can motivate the public to actively participate and enhance their hedonistic experience.

Thirdly, the cultural tourism industry should realize intellectual property linkages to classic culture. Museums with cultural value can guide the public to their long histories, so they can discover the beauty of cultural relics and embrace traditional culture as a window to history and civilization. Cultural intellectual property usually refers to the connection and integration between cultures, with strong recognition, a loyal fan base, innovative cross-border cooperation, and strong realizing ability (Jiang et al., 2023). Virtual museums are not just about displaying collections in virtual spaces, they are also about using digital technologies to showcase cultural connotations. These practices can effectively awaken the various cultural resources that lie dormant in museums and enhance the cultural identity of the public.

### 5.3. Limitations

This paper empirically analyses the relationship between the digital museuming experience, perceived value, cultural identity and willingness to visit on site in the process of digital museuming. However, there are some limitations. For example, the experience of digital museuming is a dynamic process, and this paper uses questionnaires that rely on users’ memories of digital museuming rather than experimental methods to capture real-time psychological responses. The deep integration of museums and virtual reality technology can be further explored by, firstly, considering more dimensions of the digital museuming experience and, secondly, selecting more museum cases for comparative study. In conclusion, the impact of the digital museuming experience on willingness to visit on site is a new research topic that can be studied by taking a multidisciplinary perspective on the digital communication of museums.

## Data availability statement

The raw data supporting the conclusions of this article will be made available by the authors, without undue reservation.

## Ethics statement

The studies involving human participants were reviewed and approved by Academic Ethics and Morals Committee of Zhengzhou University. The patients/participants provided their written informed consent to participate in this study.

## Author contributions

YD, XZ, JQ and BoZ contributed to conception and design of the study. XZ organized the database. BeZ and XZ performed the statistical analysis. YD and XZ wrote the first draft of the manuscript. BoZ, BeZ, and YD wrote sections of the manuscript. All authors contributed to manuscript revision, read, and approved the submitted version.

## Funding

This work was supported by Major Project of Philosophy and Social Science Research in Henan Higher Education (No. 2022-JCZD-24), Special Project for the Construction of Journalism and Communication Discipline at Zhengzhou University in Henan Province (No. 21XKJS007), China Postdoctoral Science Foundation Grant (No. 2022 M722890), and Henan Philosophy and Social Science Planning Project (No. 2021CXW031).

## Conflict of interest

The authors declare that the research was conducted in the absence of any commercial or financial relationships that could be construed as a potential conflict of interest.

## Publisher’s note

All claims expressed in this article are solely those of the authors and do not necessarily represent those of their affiliated organizations, or those of the publisher, the editors and the reviewers. Any product that may be evaluated in this article, or claim that may be made by its manufacturer, is not guaranteed or endorsed by the publisher.

## References

[ref1] Agarwal R. Karahanna E. (2000). Time flies when you're having fun: cognitive absorption and beliefs about information technology usage. MIS Q. 24, 665–694. doi: 10.2307/3250951

[ref2] Agostino D. Arnaboldi M. Lampis A. (2020). Italian state museums during the COVID-19 crisis: from onsite closure to online openness. Museum Manag. Curator. 35, 362–372. doi: 10.1080/09647775.2020.1790029

[ref3] Alba J. W. Hutchinson J. W. (1987). Dimensions of consumer expertise. J. Consum. Res. 13, 411–454. doi: 10.1086/209080

[ref4] Altugan A. S. (2015). The relationship between cultural identity and learning. Procedia Soc. Behav. Sci. 186, 1159–1162. doi: 10.1016/j.sbspro.2015.04.161

[ref5] Art China. (2020). Digital museuming Dunhuang. Amazing in the Palm: an immersive experience through the millennia. eds. Xu, B. Art China. Available online at: http://art.china.cn/txt/2020-04/15/content_41123756.shtml

[ref6] Aurindo M. J. Machado C. (2016). MUVITUR® (virtual museum of tourism): a new approach to tourism history. J. Tour. Hist. 8, 300–309. doi: 10.1080/1755182X.2017.1288763

[ref7] Babin B. J. Darden W. R. Griffin M. (1994). Work and/or fun: measuring hedonic and utilitarian shopping value. J. Consum. Res. 20, 644–656. doi: 10.1086/209376

[ref8] Chen J. Becken S. Stantic B. (2022). Travel bubbles to maintain safe space for international travel during crisis–emotions reflected in twitter posts. Curr. Issue Tour. 1, 1–15. doi: 10.1080/13683500.2022.2089546

[ref9] Chen J. Liao J. (2022). Antecedents of viewers’ live streaming watching: a perspective of social presence theory. Front. Psychol. 13:9629. doi: 10.3389/fpsyg.2022.839629, PMID: 35432106 PMC9008234

[ref10] Cheng L. K. Huang H. L. (2022). Virtual tourism atmospheres: the effects of pleasure, arousal, and dominance on the acceptance of virtual tourism. J. Hosp. Tour. Manag. 53, 143–152. doi: 10.1016/j.jhtm.2022.10.002

[ref11] Choi B. Kim J. (2021). Changes and challenges in museum management after the COVID-19 pandemic. J. Open Innov. 7:148. doi: 10.3390/joitmc7020148

[ref12] Corstorphine E. (2006). Cognitive–emotional–behavioral therapy for the eating disorders: working with beliefs about emotions. Eur. Eating Disord. Rev. 14, 448–461. doi: 10.1002/erv.747

[ref13] Errichiello L. Micera R. Atzeni M. Del Chiappa G. (2019). Exploring the implications of wearable virtual reality technology for museum visitors' experience: a cluster analysis. Int. J. Tour. Res. 21, 590–605. doi: 10.1002/jtr.2283

[ref14] Farivar S. Wang F. (2022). Effective influencer marketing: a social identity perspective. J. Retail. Consum. Serv. 67:103026. doi: 10.1016/j.jretconser.2022.103026

[ref15] Flavián C. Guinalíu M. Gurrea R. (2006). The role played by perceived usability, satisfaction and consumer trust on website loyalty. Inf. Manag. 43, 1–14. doi: 10.1016/j.im.2005.01.002

[ref16] Gallarza M. G. Saura I. G. (2006). Value dimensions, perceived value, satisfaction and loyalty: an investigation of university students’ travel behavior. Tour. Manag. 27, 437–452. doi: 10.1016/j.tourman.2004.12.002

[ref17] Gao C. H. Wang R. X. Sun Z. F. (2020). Ethnic contact weakens ethnic essentialism: the mediating role of cultural identity and cultural similarity. J. Psychol. Sci. 2, 445–451. doi: 10.16719/j.cnki.1671-6981.20200226

[ref18] Güzel F. Ö. (2014). The dimensions of tour experience, emotional arousal, and post-experience behaviors: a research on Pamukkale in Turkey. Procedia Soc. Behav. Sci. 150, 521–530. doi: 10.1016/j.sbspro.2014.09.069

[ref19] Han D. (2020). Perceived value, brand identity and customer response in virtual brand Community, journal of. Bus. Econ. 9, 84–87.

[ref20] He J. Ai S. (2021). Study on impact of Daming palace National Heritage Park tourist experience on tourists’ cultural identity. Areal Res. Dev. 40, 99–108. doi: 10.3969/j.issn.1003-2363.2021.03.017

[ref21] He Z. Wu L. Li X. R. (2018). When art meets tech: the role of augmented reality in enhancing museum experiences and purchase intentions. Tour. Manag. 68, 127–139. doi: 10.1016/j.tourman.2018.03.003

[ref22] Jiang Y. Ma Z. Wang X. (2023). The impact of knowledge management on intellectual property risk prevention: analysis from China’s strategic emerging industries. J. Knowl. Manag. 27, 197–207. doi: 10.1108/JKM-03-2022-0216

[ref23] Jin N. Lee S. Lee H. (2015). The effect of experience quality on perceived value, satisfaction, image and behavioral intention of water park patrons: new versus repeat visitors. Int. J. Tour. Res. 17, 82–95. doi: 10.1002/jtr.1968

[ref24] Kang E. J. Scott N. Lee T. J. Ballantyne R. (2012). Benefits of visiting a ‘dark tourism’site: the case of the Jeju April 3rd Peace Park Korea. Tourism Management 33, 257–265. doi: 10.1016/j.tourman.2011.03.004

[ref25] Kaur H. Paruthi M. Islam J. Hollebeek L. D. (2020). The role of brand community identification and reward on consumer brand engagement and brand loyalty in virtual brand communities. Telematics Inform. 46:101321. doi: 10.1016/j.tele.2019.101321

[ref26] Keillor B. D. Hult G. T. M. Erffmeyer R. C. Babakus E. (1996). NATID: the development and application of a national identity measure for use in international marketing. J. Int. Mark. 4, 57–73. doi: 10.1177/1069031X9600400205

[ref27] Kim J.-H. Ritchie J. R. B. McCormick B. (2012). Development of a scale to measure memorable tourism experiences. J. Travel Res. 51, 12–25. doi: 10.1177/0047287510385467

[ref28] Li X. J. Li Z. R. Song C. Y. Lu W. L. Zhang Q. (2021). A study on the mechanism of virtual tourism behavior based on the theory of planned behavior. Tour. J. 36, 15–26. doi: 10.19765/j.cnki.1002-5006.2021.08.007

[ref29] Liao J. Chen K. Qi J. Li J. Yu I. Y. (2023). Creating immersive and parasocial live shopping experience for viewers: the role of streamers' interactional communication style. J. Res. Interact. Mark. 17, 140–155. doi: 10.1108/JRIM-04-2021-0114

[ref30] Luo W. Xie D. Tang Y. Zhong P. (2021). Influence of tourists’ poetry cognition and emotional evaluation on their behavioral intention. J. Hubei Univ. Arts Sci. 42, 43–51.

[ref31] Marty P. F. (2007). Museum websites and museum visitors: before and after the museum visit. Museum Manag. Curator. 22, 337–360. doi: 10.1080/09647770701757708

[ref32] Meng L. (2018). The impact of perceived value in virtual brand community on consumer behavior. J. Bus. Econ. 2, 46–49.

[ref33] Meng Y. Chu M. Y. Chiu D. K. (2022). The impact of COVID-19 on museums in the digital era: practices and challenges in Hong Kong. Hong Kong: Library Hi Tech.

[ref34] Otto J. E. Ritchie J. R. B. (1995). Exploring the quality of the service experience: a theoretical and empirical analysis. Adv. Serv. Mark. Manag. 4, 37–61. doi: 10.1016/S1067-5671(95)04018-8

[ref35] Otto J. E. Ritchie J. R. B. (1996). The service experience in tourism. Tour. Manag. 17, 165–174. doi: 10.1016/0261-5177(96)00003-9

[ref36] Pan X. Hao A. Guan C. Hsieh T. J. (2019). Affective and cognitive dimensions in cultural identity: scale development and validation. Asia Pac. J. Mark. Logist. 32, 1362–1375. doi: 10.1108/APJML-03-2019-0200

[ref37] Perez-Marcos D. (2018). Virtual reality experiences, embodiment, videogames and their dimensions in neurorehabilitation. J. Neuroeng. Rehabil. 15, 113–118. doi: 10.1186/s12984-018-0461-0, PMID: 30477527 PMC6258149

[ref38] Prayag G. Ryan C. (2012). Antecedents of tourists’ loyalty to Mauritius: the role and influence of destination image, place attachment, personal involvement, and satisfaction. J. Travel Res. 51, 342–356. doi: 10.1177/0047287511410321

[ref39] Proctor N. (2011). The Google art project: a new generation of museums on the web? Curator Museum J. 54, 215–221. doi: 10.1111/j.2151-6952.2011.00083.x

[ref40] Rafferty E. Pad B. (2017). Better together: a holistic approach to creating a digital preservation policy in an art museum art documentation. J. Art Lib. Soc. North America 36, 149–162. doi: 10.1086/691378

[ref41] Russo A. Watkins J. Kelly L. Chan S. (2006). How will social media affect museum communication?. Proceedings: Nordic Digital Excellence in Museums (NORDIC 06), 1–4. Available at: http://eprints.qut.edu.au

[ref42] Samaroudi M. Echavarria K. R. Perry L. (2020). Heritage in lockdown: digital provision of memory institutions in the UK and US of America during the COVID-19 pandemic. Museum Manag. Curator. 35, 337–361. doi: 10.1080/09647775.2020.1810483

[ref43] Sanchez-Vives M. V. Slater M. (2005). From presence to consciousness through virtual reality. Nat. Rev. Neurosci. 6, 332–339. doi: 10.1038/nrn1651, PMID: 15803164

[ref44] Schubert T. W. (2009). A new conception of spatial presence: once again, with feeling. Commun. Theory 19, 161–187. doi: 10.1111/j.1468-2885.2009.01340.x

[ref45] Shipps B. Phillips B. (2013). Social networks, interactivity and satisfaction: assessing socio-technical behavioral factors as an extension to technology acceptance. J. Theor. Appl. Electron. Commer. Res. 8, 7–8. doi: 10.4067/S0718-18762013000100004

[ref46] Skadberg Y. X. Kimmel J. R. (2004). Visitors’ flow experience while browsing a web site: its measurement, contributing factors and consequences. Comput. Hum. Behav. 20, 403–422. doi: 10.1016/S0747-5632(03)00050-5

[ref47] Thomas W. A. Carey S. (2005). “Actual/virtual visits: what are the links? Museums and the web 2005” in Proceedings from the international conference, vol. 4 (Vancouver, British Columbia: Archives & Museum Informatics), 13–17.

[ref48] Tian D. Wang Q. Law R. Zhang M. (2020). Influence of cultural identity on tourists’ authenticity perception, tourist satisfaction, and traveler loyalty. Sustainability 12:6344. doi: 10.3390/su12166344

[ref49] Trunfio M. Campana S. (2020). A visitors’ experience model for mixed reality in the museum. Curr. Issue Tour. 23, 1053–1058. doi: 10.1080/13683500.2019.1586847

[ref50] United Nations Educational, Scientific, and Cultural Organization (UNESCO) (2020). Museums around the world in the face of COVID-19. Available online at: https://unesdoc.unesco.org/ark:/48223/pf0000373530

[ref51] Wu H. C. Ai C. H. Cheng C. C. (2020). Virtual reality experiences, attachment and experiential outcomes in tourism. Tour. Rev. 75, 481–495. doi: 10.1108/TR-06-2019-0205

[ref52] Xiao Z. Deling Y. (2016). The expansion of art through cultural postproduction in online virtual museums, Proceedings of the symposium on VR culture and heritage 2, 51–57. doi: 10.1145/3014027.3017433

[ref53] Xinhua News Agency. (2020). Digital museuming at home. eds. Wang X. Wei B. Xinhua News Agency. Available online at: https://baijiahao.baidu.com/s?id=1659319657381729107

[ref54] Xu X. Huang D. Shang X. (2021). Social presence or physical presence? Determinants of purchasing behavior in tourism live-streamed shopping. Tour. Manag. Perspect. 40:100917. doi: 10.1016/j.tmp.2021.100917

[ref55] Zahorik P. Jenison R. L. (1998). Presence as being-in-the-world. Presence 7, 78–89. doi: 10.1162/105474698565541

[ref56] Zeithaml V. A. (1988). Consumer perceptions of price, quality, and value: a means-end model and synthesis of evidence. J. Mark. 52, 2–22. doi: 10.1177/002224298805200302

[ref57] Zeng R. Zhu D. (2021). Fear of evaluation and online self-disclosure on WeChat: moderating effects of protective face orientation. Front. Psychol. 12:722. doi: 10.3389/fpsyg.2021.530722, PMID: 34512427 PMC8424039

[ref58] Zhang S. N. Ruan W. Q. Yang T. T. (2021). National identity construction in cultural and creative tourism: the double mediators of implicit cultural memory and explicit cultural learning. SAGE Open 11:789. doi: 10.1177/21582440211040789

[ref59] Zhang W. Z. Xu Y. F. (2021). The sense of presence and individual experience difference in virtual environment. J. Fujian Med. Univ. 22, 22–28.

[ref60] Zheng S. Wu M. Liao J. (2022). The impact of destination live streaming on viewers’ travel intention. Curr. Issue Tour. 26, 184–198. doi: 10.1080/13683500.2022.2117594

[ref61] Zhou M. Zhu J. Zhou Z. Zhou H. Ji G. (2022). Cognitive bias toward the internet: the causes of adolescents’ internet addiction under parents’ self-affirmation consciousness. Front. Psychol. 13:1473. doi: 10.3389/fpsyg.2022.891473, PMID: 35978789 PMC9376473

