# Supplementary material for: From digital museuming to on-site visiting: The mediation of cultural identity and perceived value
Source: Front Psychol. 2023 Mar 22;14:1111917. doi: 10.3389/fpsyg.2023.1111917 (PMC10074853; doi:10.3389/fpsyg.2023.1111917)
Supplement: Supplementary file 1 [file Data_Sheet_1.docx]

#### Appendix

#### Supplementary Table 1 Multiple mediation effects test

| Variable | Hypothesized paths | R^2^ | F | LLCI | ULCI | t | P |
| --- | --- | --- | --- | --- | --- | --- | --- |
| Cognitive immersion experience | PIE→PV | 0.305 | 46.513 | 0.440 | 0.591 | 13.404 | 0 |
|  | PIE→CI | 0.487 | 80.360 | 0.364 | 0.516 | 11.390 | 0 |
|  | PV→CI |  |  | 0.227 | 0.388 | 7.514 | 0 |
|  | PIE→WVS | 0.547 | 84.935 | 0.118 | 0.298 | 4.534 | 0 |
|  | PV→WVS |  |  | 0.200 | 0.378 | 6.404 | 0 |
|  | CI→WVS |  |  | 0.326 | 0.524 | 8.441 | 0 |
|  | PIE→WVS | 0.365 | 61.007 | 0.533 | 0.689 | 15.418 | 0 |
| Interactive experience | IE→PV | 0.395 | 69.112 | 0.544 | 0.692 | 16.405 | 0 |
|  | IE→CI | 0.411 | 58.957 | 0.264 | 0.447 | 7.617 | 0 |
|  | PV→CI |  |  | 0.246 | 0.431 | 7.205 | 0 |
|  | IE→WVS | 0.545 | 84.360 | 0.115 | 0.304 | 4.349 | 0 |
|  | PV→WVS |  |  | 0.160 | 0.350 | 5.291 | 0 |
|  | CI→WVS |  |  | 0.372 | 0.557 | 9.872 | 0 |
|  | IE→WVS | 0.352 | 57.534 | 0.547 | 0.712 | 14.965 | 0 |
| Available Experience | AE→PV | 0.306 | 46.750 | 0.450 | 0.604 | 13.439 | 0 |
|  | AE→CI | 0.428 | 63.222 | 0.273 | 0.437 | 8.503 | 0 |
|  | PV→CI |  |  | 0.276 | 0.446 | 8.337 | 0 |
|  | AE→WVS | 0.539 | 82.360 | 0.075 | 0.250 | 3.635 | 0 |
|  | PV→WVS |  |  | 0.202 | 0.383 | 6.331 | 0 |
|  | CI→WVS |  |  | 0.375 | 0.564 | 9.755 | 0 |
|  | AE→WVS | 0.309 | 47.297 | 0.489 | 0.655 | 13.542 | 0 |
| Hedonic experience | HE→PV | 0.272 | 39.567 | 0.426 | 0.587 | 12.336 | 0 |
|  | HE→CI | 0.438 | 65.900 | 0.291 | 0.453 | 9.016 | 0 |
|  | PV→CI |  |  | 0.286 | 0.450 | 8.788 | 0 |
|  | HE→WVS | 0.528 | 78.515 | -0.021 | 0.158 | 1.502 | 0.134 |
|  | PV→WVS |  |  | 0.237 | 0.418 | 7.125 | 0 |
|  | CI→WVS |  |  | 0.410 | 0.603 | 10.301 | 0 |
|  | HE→WVS | 0.243 | 34.099 | 0.428 | 0.606 | 11.450 | 0 |

#### Supplementary Table 2 Multi-mediated effect test

|  |  | Effect Value | S.E. | LLCI | ULCI | Relative effect value |
| --- | --- | --- | --- | --- | --- | --- |
| Cognitive immersion experience | Total effect | 0.6111 | 0.0396 | 0.5332 | 0.6891 |  |
|  | Direct effect | 0.2076 | 0.0458 | 0.1176 | 0.2976 | 0.340 |
|  | Indirect total effect | 0.4036 | 0.0486 | 0.3107 | 0.5033 | 0.660 |
|  | Ind1 | 0.1490 | 0.0373 | 0.0802 | 0.2273 |  |
|  | Ind2 | 0.1872 | 0.0395 | 0.1140 | 0.2669 |  |
|  | Ind3 | 0.0674 | 0.0187 | 0.0366 | 0.1090 |  |
| Interactive experience | Total effect | 0.6296 | 0.0421 | 0.5469 | 0.7122 |  |
|  | Direct effect | 0.2094 | 0.0482 | 0.1148 | 0.3041 | 0.333 |
|  | Indirect total effect | 0.4201 | 0.0505 | 0.3231 | 0.5187 | 0.667 |
|  | Ind4 | 0.1578 | 0.0451 | 0.0733 | 0.2518 |  |
|  | Ind5 | 0.1651 | 0.0378 | 0.0971 | 0.2433 |  |
|  | Ind6 | 0.0973 | 0.0246 | 0.0543 | 0.1493 |  |
| Available Experience | Total effect | 0.5721 | 0.0422 | 0.4891 | 0.6552 |  |
|  | Direct effect | 0.1624 | 0.0447 | 0.0746 | 0.2502 | 0.284 |
|  | Indirect total effect | 0.4098 | 0.0458 | 0.323 | 0.5029 | 0.716 |
|  | Ind7 | 0.1541 | 0.0394 | 0.0812 | 0.2365 |  |
|  | Ind8 | 0.1665 | 0.0350 | 0.1036 | 0.2384 |  |
|  | Ind9 | 0.0892 | 0.0217 | 0.0513 | 0.1365 |  |
| Hedonic experience | Total effect | 0.5171 | 0.0452 | 0.4283 | 0.6059 |  |
|  | Direct effect | 0.0684 | 0.0455 | -0.0211 | 0.1579 | 0.132 |
|  | Indirect total effect | 0.4487 | 0.0498 | 0.3546 | 0.5510 | 0.868 |
|  | Ind10 | 0.1660 | 0.0382 | 0.0950 | 0.2455 |  |
|  | Ind11 | 0.1883 | 0.0384 | 0.1195 | 0.2694 |  |
|  | Ind12 | 0.0943 | 0.0222 | 0.0562 | 0.1420 |  |
